# Alveolar bone defects in Indian dry skulls: Prevalence of fenestration and dehiscence

**DOI:** 10.6026/973206300220834

**Published:** 2026-02-28

**Authors:** Aniket Meghawat, Usha Bosak, Kshiti Bhushan Pandaw, Aditya Pawar, Ashwini Dhopte

**Affiliations:** 1 Department of Periodontics, Sharad Pawar Dental College and Hospital, Wardha, Maharashtra, India; 2 Department of Periodontics, Aakanksha Dental Care and Implant Center, Raipur, Chattisgarh, India; 3 Department of Oral Medicine and Radiology, Chhattisgarh Dental College and Research Institute, Chhattisgarh, India

**Keywords:** Alveolar process, bone defects, dehiscence, fenestration

## Abstract

Bone dehiscence and fenestration are alveolar bone defects that can adversely affect periodontal health and complicate dental surgical procedures, yet data on their prevalence in the Indian population remain limited. Therefore, it is of interest to evaluate the occurrence and distribution of dehiscence and fenestration in relation to specific teeth using 200 maxillae and 200 mandibles from Indian dry skulls obtained from Maitri Dental College and Research Centre and other dental colleges in India. Visual examination revealed that canines, first premolars and incisors were the teeth most commonly associated with these defects. Dehiscence was observed more frequently than fenestration and both defects were predominantly bilateral in distribution. Careful preoperative assessment of these defects is essential, as their presence may influence treatment planning and increase the risk of complications during periodontal and dental surgical procedures.

## Background:

Fenestration can be defined as isolated areas in which the root is denuded of bone and the root surface is covered only by periosteal and overlying gingiva. In these areas, the marginal bone is intact. A dehiscence is loss of alveolar bone on the facial aspect of a tooth that leaves a root-exposed defect from cemento-enamel junction apically. The etiology of these defects is unknown. However, microscopic evidence revealed lacunar resorption at the margins. Predisposing factors comprised of prominent root contours, malposition and labial protrusion of the root in combination with a thin bony plate. Fenestration and dehiscence are paramount as they may complicate the outcome of periodontal surgery. These defects often occur where a tooth during eruption is displaced out of the arch and are more frequent over anterior teeth. The root in such defects is covered only by a connective tissue attachment and overlying mucosa [1, 2]. The partial-thickness flap and apically displaced flap may be necessary in case of dehiscence or fenestrations [1]. Therefore, it is of interest to evaluate the prevalence of fenestration and dehiscence in central Indian dry skulls.

## Materials and Methods:

This research was performed on dried human skulls belonging to the Indian population. The inclusion criteria with the date of death placed in the interval of 1999-2019. Skulls should be dentate ones, with complete dentition or a reduced number of missing teeth.

This research was performed on 200 Maxilla and 200 Mandible. It was done by visual examination of the dry skulls;

[1] According to fenestration and dehiscence found in right and left side of maxilla and mandible

[2] According to the tooth region

[3] According to anterior and posterior region of jaw.

## Results:

Table 1 show that fenestration is most frequently found in left maxillary [46 (23%)] followed by right maxillary [35 (17.5%)]. This shows a bilateral fenestration in maxillary is common finding. When evaluating mandibular defects fenestration was seen to be on [7 (3.5%)] right mandible and [0 (0%)] in left mandible making it an isolated finding rather than a bilateral one. Dehiscence in most frequently found in right maxillary and mandible [41 (20.5%) and 48 (24%)] followed by left maxillary and mandible [39 (19.5%) and 45 (22.5%)]. This shows a bilateral dehiscence in maxillary is common finding. Table 2 and Table 3 shows that fenestration is most frequently found in canine [43 (10.75%)] followed by 1st premolar [30 (7.5%) and 26 (6.5%)] in maxillary and in mandible more frequently found in lateral incisors [16 (4%)] followed by canine [10 (2.5%)]. Dehiscence is more frequently found in maxillary canine [124 (31%)] followed by central incisors [56 (14%) and 52 (13%)] and in mandible canine [96 (24%)] followed by lateral incisors [55 (13.75%] and 1st premolar [42 (10.5%)]. Table 4 shows that fenestration is most frequently found in anterior region of maxilla [78 (6.5%)] and mandible [29 (2.41%)]. Table 5 shows that dehiscence is more frequently found in anterior region of maxilla [224 (18.67%)] and mandible [190 (15.87%)]. Prevalence of fenestrations in maxilla is as follow: (ascending order) first molar < second premolar < lateral incisor < first premolar < canine and in mandibular arch it is second molar < first premolar < central incisors < canine < lateral incisors. Prevalence of dehiscence in maxillary in ascending order second premolar < first molar < lateral incisor < first premolar < central incisor < canine and in mandibular first molar < central incisor < first premolar < lateral incisors < canine.

## Discussion:

There is broad agreement in previous literature that fenestration represents a localized or circumscribed defect of the alveolar bone plate resulting in exposure of the underlying root surface; however, such uniformity is lacking with respect to the definition of dehiscence. The present study demonstrated that alveolar dehiscences and fenestrations are common findings in the Indian population. Definitions of dehiscence reported in the literature vary widely, ranging from the absence of alveolar cortical bone exceeding half the root length to any deficiency of alveolar bone leading to denudation of the root surface [3]. The maxillary and mandibular distribution of defects observed in the present study was generally consistent with earlier investigations, wherein fenestrations were more frequently identified in the maxilla, while dehiscences predominated in the mandible. Dehiscences were most commonly associated with maxillary canines and central incisors, whereas fenestrations were frequently observed in maxillary lateral incisors and canines. These findings, however, contrast with reports suggesting a comparable prevalence of dehiscences and fenestrations in the mandible. The prevalence and distribution patterns identified in this study differ from those reported in earlier skeletal analyses, which documented lower overall prevalence rates and differing tooth-specific associations [4, 5]. A progressive decline in the prevalence of both dehiscences and fenestrations with increasing age was observed in the present study, a finding consistent with earlier observations [4]. This trend may be attributed to increased tooth loss in older individuals, thereby reducing the number of teeth at risk. Additionally, fenestrations present in younger individuals may transform into dehiscences over time due to progressive alveolar bone loss associated with periodontitis, endodontic pathology, or trauma. Interdental bone loss occurring in younger subjects may further alter the morphology of dehiscence defects, rendering them less recognizable in older individuals. Although the contribution of post-mortem artifacts to the presence of alveolar defects cannot be completely excluded, specimens exhibiting visible damage were excluded from the analysis and only adult skulls were included to minimize this limitation. The present findings revealed that dehiscences and fenestrations were most prevalent in canines, which is in agreement with previous observations [2]. While dehiscence was more frequently observed in incisors in the present study, this finding contrasts with earlier reports that documented a higher prevalence in canines [3]. Such discrepancies may be attributed to genetic and morphological variations between regional populations within India. Excessive occlusal forces have been proposed as a contributing etiological factor in the development of fenestrations and dehiscences [6]. Some investigations have reported evidence of attrition suggestive of excessive occlusal loading in teeth exhibiting fenestrations [7], whereas others have failed to demonstrate a consistent association between occlusal wear and these alveolar defects [8]. Gingival recession, characterized by apical migration of the gingival margin with exposure of the root surface, has also been associated with fenestration and dehiscence, among other contributing factors [7, 9, 10]. The radiographic detection of fenestrations and dehiscences remains limited due to superimposition of the defect over the root surface. Clinical palpation of root prominence and bone sounding performed under local anesthesia has been recommended as adjunctive diagnostic methods [11, 12]. Advances in three-dimensional imaging, particularly cone-beam computed tomography (CBCT), have significantly enhanced the detection of alveolar defects that may otherwise remain undiagnosed. CBCT has been shown to aid in the accurate diagnosis of apical fenestrations that were previously misinterpreted as persistent apical pathology [13]. Excessive labial positioning of teeth has been reported to result in alveolar bone denudation extending approximately 1-1.5 mm apical to the cemento- enamel junction [11]. Root fractures also represent an important predisposing factor for alveolar defects. Dehiscences are commonly associated with vertical root fractures, particularly involving the buccal cortical plate, whereas fenestrations may occur when fractures are confined to the root without coronal or apical extension [14, 15].

## Conclusion:

Canines and first premolars were the teeth most commonly associated with alveolar dehiscence, which occurred more frequently than fenestration in the examined skulls. Both defects showed a predominantly bilateral distribution, with canines, first premolars and incisors being most frequently involved. Careful evaluation of these defects before dental and periodontal surgical procedures is essential, as their presence may adversely influence surgical outcomes.

## Clinical significance:

The presence of alveolar bone dehiscence and fenestration has significant implications for various dental procedures. Their identification is crucial to prevent potential complications in periodontal and surgical treatments:

[1] Periodontal Surgery: These defects can complicate flap surgeries, grafting procedures and regenerative treatments. Inadequate bone coverage may lead to further recession and compromised healing.

[2] Implant Placement: Bone dehiscence and fenestration may affect implant stability, requiring bone grafting or guided bone regeneration to ensure successful osseointegration.

[3] Orthodontic Treatment: Tooth movement in the presence of dehiscence can lead to increased root exposure and gingival recession, necessitating careful force application and monitoring.

[4] Diagnosis and Treatment Planning: Recognizing these defects early enables clinicians to modify treatment plans, adopt preventive measures and avoid iatrogenic damage, ultimately improving patient outcomes.

## List of abbreviations:

[1] CBCT - Cone Beam Computed Tomography

[2] CEJ - Cemento-Enamel Junction

[3] PDL - Periodontal Ligament

[4] RCT - Root Canal Treatment

[5] GTR - Guided Tissue Regeneration

[6] GBR - Guided Bone Regeneration

[7] BMD - Bone Mineral Density

[8] OHI - Oral Hygiene Instructions

[9] mm - Millimeter

[10] % - Percentage

## Figures and Tables

**Table 1:** Frequency of dehiscence and fenestration found in right and left maxillary and mandible

**Fenestration**	**Right Maxilla**	**Right Mandible**	**Left Maxilla**	**Left Mandible**
0	148(74%)	190(95%)	144(72%)	190(95%)
1	35(17.5%)	7(3.5%)	46(23%)	0(0%)
2	13(6.5%)	3(1.5%)	6(3%)	10(5%)
3	4(2%)	0%	4(2%)	
Total	200(100%)	200(100%)	200(100%)	200(100%)
**Dehiscence**	**Right Maxilla**	**Right Mandible**	**Left Maxilla**	**Left Mandible**
0	120(60%)	112(56%)	126(63%)	133(66.5%)
1	41(20.5%)	48(24%)	39(19.5%)	45(22.5%)
2	11(5.5%)	16(8%)	13(6.5%)	19(9.5%)
3	13(6.5%)	10(5%)	11(5.5%)	3(1.5%)
4	7(3.5%)	14(7%)	7(3.5%)	
5	2(1%)		4(2%)	
6	4(2%)			
7	2(1%)			
Total	200(100%)	200(100%)	200(100%)	200(100%)

**Table 2:** Frequency of dehiscences and fenestration in individual teeth of maxillary

**Teeth**	**Total Number Of Teeth**	**Present Fenestration**	**Percentage**
Third Molar	400	2	0.5
Second Molar	400	4	1
First Molar	400	17	4.25
Second Premolar	400	19	4.75
First Premolar	400	30	7.5
Canine	400	43	10.75
Lateral Incisor	400	26	6.5
Central Incisor	400	8	2
	3200	149	4.65
**Teeth**	**Total Number of Teeth**	**Dehiscences**	**Percentage**
Third Molar	400	15	3.75
Second Molar	400	13	3.25
First Molar	400	39	9.75
Second Premolar	400	32	8
First Premolar	400	52	13
Canine	400	124	31
Lateral Incisor	400	43	10.75
Central Incisor	400	56	14
Total	3200	374	11.69

**Table 3:** Frequency of dehiscences and fenestration found in individual teeth in mandible

**Teeth**	**Total Number Of Teeth**	**Fenestration**	**Percentage**
Third Molar	400	0	0
Second Molar	400	3	0.75
First Molar	400	0	0
Second Premolar	400	0	0
First Premolar	400	3	0.75
Canine	400	10	2.5
Lateral Incisors	400	16	4
Central Incisors	400	4	1
Total	3200	36	1.125
**Teeth**	**Total Number of Teeth**	**Present Dehiscence**	**Percentage**
Third Molar	400	0	0
Second Molar	400	0	0
First Molar	400	13	3.25
Second Premolar	400	0	0
First Premolar	400	42	10.5
Canine	400	96	24
Lateral Incisors	400	55	13.75
Central Incisors	400	38	9.5
Total	3200	244	7.625

**Table 4:** Fenestration found in anterior and posterior region of maxillary and mandible

	**Maxilla**		**Mandible**	
**S.No**	**Anterior**	**Posterior**	**Anterior**	**Posterior**
Total Number Tooth Examined	1200	1200	1200	1200
Number of Fenestration Defects	78	43	29	4
Percentage of Defects	6.5	3.58	2.41	0.33
P Value	0.001		<0.0001	

**Table 5:** Dehiscence found in anterior and posterior region of maxillary and mandible

	**Maxilla**		**Mandible**	
**S.NO**	**Anterior**	**Posterior**	**Anterior**	**Posterior**
Total Number Tooth Examined	1200	1200	1200	1200
Number of Dehiscence Defects	224	91	190	33
Percentage of Defects	18.67	7.58	15.83	2.75
P value	<0.0001 HS		<0.0001 hs	

## References

[1] Kajan ZD (2020). Dent Res J (Isfahan)..

[2] Nalbantoglu AM, Yanik D (2023). Aust Dent J..

[3] Choi JY (2020). Prog Orthod..

[4] Sun L (2022). Clin Oral Investig..

[5] Luo N (2024). Orthod Craniofac Res..

[6] Sheng Y (2020). J Orofac Orthop..

[7] Ferrari CH (2024). Methods Protoc..

[8] Van Leeuwen BJ (2022). Clin Oral Investig..

[9] Jing WD (2021). Am J Orthod Dentofacial Orthop..

[10] Moon HW (2020). Angle Orthod..

[11] Movahhedian N (2020). Cleft Palate Craniofac J..

[12] Dominiak M (2021). Ann Anat..

[13] Han S (2024). Am J Orthod Dentofacial Orthop..

[14] Hu X (2020). Am J Orthod Dentofacial Orthop..

[15] Gaffuri F (2021). Saudi Dent J..

